# Supporting the Aspecific Physiological Defenses of Upper Airways against Emerging SARS-CoV-2 Variants

**DOI:** 10.3390/pathogens12020211

**Published:** 2023-01-29

**Authors:** Luca Cegolon, Giuseppe Mastrangelo, Saverio Bellizzi, Francesca Larese Filon, Cristiano Salata

**Affiliations:** 1 Department of Medical, Surgical & Health Sciences, University of Trieste, 34129 Trieste, Italy; 2 Occupational Medicine Unit, University Health Agency Giuliano-Isontina (ASUGI), 34129 Trieste, Italy; 3 Senior Researcher, University of Padua, 35121 Padua, Italy; 4 Independent Researcher, 1202 Geneva, Switzerland; 5 Department of Molecular Medicine, University of Padua, 35121 Padua, Italy

The rapid rollout of COVID-19 vaccines in 2021 sparked general optimism toward controlling the severe form of the disease, preventing hospitalizations and COVID-19-associated mortality, and the transmissibility of SARS-CoV-2 infection [1,2,3]. However, due to their high frequency of mutation [4], human coronaviruses are known to cause re-infections regardless of pre-existing humoral immunity [5,6]. 

Since December 2021, the Omicron variant, whose spike protein highly diverges from previous viral strains, spread aggressively worldwide, also among vaccinated individuals, rapidly becoming the dominant variant by January 2022 [5,7]. Although characterized by a clinical presentation of flu-like symptoms lasting a few days, with a case fatality rate < 0.01%, a hospitalization rate of 0.3% and a short length of hospital stay, Omicron raised immediate concerns for the high risk of vaccine failure due to the evasion of neutralizing antibody responses [8,9,10]. The effectiveness of COVID-19 vaccines progressively decreased following the Delta wave and SARS-CoV-2 re-infections, which were almost non-existent before the Omicron transmission period, started to surge from December 2021 onward [5,6,11,12,13]. 

The mild clinical presentation of Omicron progressively shifted public health attention from the containment of morbidity to the prevention and control of SARS-CoV-2 infection. With a rapidly mutating virus heading toward become endemic, herd immunity by mass vaccination proved ineffective yet costly for providing long-standing protection against community transmission of SARS-CoV-2. However, harmless treatments that are easy to administer in outpatient settings were immediately indicated to be critically important since the early phase of the pandemic for controlling the transmissibility of SARS-CoV-2 from patients affected by mild–moderate disease [14,15,16].

Off-label therapies have been recommended or tested before and even after COVID-19 vaccines became available to tackle the saturation of hospital beds and shortage of healthcare force. Several studies, conducted predominantly in vitro, tested the efficacy of different active compounds in the early phase of infection, as post-exposure prophylaxis to reduce viral shedding time (VST) and mitigate the disease progression [14,17,18]. 

SARS-CoV-2 enters the human body predominantly through the nasal cavity, where the virus first infects the multi-ciliated cells of the nasopharynx or the sustentacular cells of the nasal olfactory mucosa [19]. Aerosol modelling suggests that the highest multiplicity of infection of SARS-CoV-2 per unit tissue surface area may occur in the nasal cavity, since its local mucosa presents the highest expression of ACE-2 receptor, the primary port of entry for the virus into target cells [4]. The ACE-2 receptor is also reportedly expressed in oral gingival epithelia and salivary glands, making the oral cavity a relevant viral reservoir, with saliva contributing to environmental dissemination of SARS-CoV-2 via aerosol droplets formed by talking, coughing, or breathing [20]. Nonetheless, since the early phase of SARS-CoV-2 infection, higher viral loads have been detected in the nose compared to the rest of the respiratory system, both in symptomatic and asymptomatic patients, designating the nasal cavity as a priority target for treatments that aim to prevent the transmissibility of the virus [4,18,19,21].

A systematic review and meta-analysis of 33 published studies (11 in vivo and 22 in vitro) investigated the virucidal efficacy of various compounds as mouth rinses and nasal sprays to reduce the salivary load of SARS-CoV-2 [22]. Povidone–iodine oral and nasal preparations exhibited effective virucidal activity, reducing SARS-CoV-2 loads both in vivo and in vitro. In particular, povidone–iodine was associated with the highest Log10 reduction value (LRV = 2.938; *p* = 0.0005) in vitro, followed by cetylpyridinium chloride (LRV = 2.907; *p* = 0.009). Mouthwashes with 0.07% cetylpyridinium chloride completely inactivated different SARS-CoV-2 variants (USA-WA1/2020, Alpha, Beta, Gamma, Delta) up to the limit of detection in suspension assays [20]. Povidone–iodine is a recognized anti-septic commonly used to disinfect surgical wounds, whereas the virucidal activity of cetylpyridinium chloride is linked to disruption of the lipid envelope of SARS-CoV-2 [20]. However, whilst povidone–iodine was effective both in vitro and in vivo, the evidence for the virucidal activity of cetylpyridinium chloride is still inconclusive due to a limited number of patients involved in the respective clinical study (N = 11) [22].

Following povidone–iodine, chlorhexidine was the most efficacious intervention used to reduce SARS-CoV-2 salivary viral load in vivo, with a mean difference in viral load of 72% for the former versus 86% for the latter [22]. However, the efficacy of 0.2% chlorhexidine was not confirmed in vitro. Chlorhexidine is a cationic surfactant and synthetic biguanide with broad-spectrum antimicrobial activity, effective against a number of pathogens, including herpes, influenza, parainfluenza, and hepatitis B [23]. The in vivo efficacy of chlorexidine is explained by its cationic nature, which allows it to stay for hours on surfaces of the oral cavity, thereby causing long-lasting virucidal effects. By contrast, the short contact time in experiments in vitro interferes with the virucidal activity of chlorhexidine [23].

Another compound tested against SAR-CoV-2, both in vitro and in vivo, is hydrogen peroxide, an antiseptic solution yielding hydroxyl free radicals reacting against membrane lipids and other essential cell components of micro-organisms [20,24]. It was suggested that 1% hydrogen peroxide would be more convenient than other formulations to reducing the salivary load of SARS-CoV-2, since the virus is vulnerable to oxidation in the oral environment. However, a hydrogen peroxide oral rinse was not more effective than other formulations in reducing the salivary load of SARS-CoV-2, both in vivo and in vitro (35%; LRV = 0.969) [18].

Further inhaling agents proposed against SARS-CoV-2 during the course of the pandemic included alcohol-based preparations and acetic acid [18,25]. Ethanol at a concentration >30% effectively inactivates SARS-CoV-2, but its biological tolerability may be a limitation in topical nasal applications, especially for pregnant women and children, with the USA Centre for Disease Prevention and Control (CDC) recommending alcohol-based sanitizers only for hand and fomites hygiene [18,19,20]. Acetic acid is instead a commonly available disinfectant, which effectively disrupts the viral envelope, thereby inhibiting viral transmission [25,26]. Aerosoled acetic acid was tested in a clinical trial on 29 patients: 14 receiving off-label hydroxychloroquine plus lopinavir/ritonavir versus 15 patients treated with hydroxychloroquine only combined with the inhalation of acetic acid disinfectant at a 0.34% concentration. A questionnaire-based evaluation of symptoms was performed 15 days after the administration of acetic acid in both groups. Although the improvement of symptoms was twice as high in patients treated with acetic acid and side effects were not recorded, the statistics were too small to reach a conclusion and recommend acetic acid to treat mild–moderate COVID-19 [27].

Whilst emerging evidence from in vivo studies using hydrogen peroxide, cetylpyridinium chloride, and various other active agents remains inconclusive, povidone–iodine and chlorhexidine mouth rinses are confirmed to be the most efficacious clinical interventions to reduce the oral load of SARS-CoV-2, regardless of their concentration. Routine use of mouth rinses of povidone–iodine and chlorhexidine in asymptomatic or uninfected individuals may therefore greatly contribute to the containment of VST in patients infected by SARS-CoV-2, especially in health care settings [21]. 

However, all compounds mentioned above, including povidone–iodine and chlorhexidine, are not physiological substances, and thus tolerability in real life may be an issue, especially in routinely administered nasal formulations. For instance, hypothyroidism has been linked to exposure to povidone–iodine antiseptics in neonates, and transient hyper-thyrotropinemia was reported in infants whose mothers were exposed to povidone–iodine as a skin disinfectant [18,28,29,30]. Furthermore, nasal irrigation with povidone–iodine may induce sneezing, paradoxically increasing the spread of aerosolized viral particles, and a chlorhexidine mouth rinse might also induce coughing, increasing the risk of viral shedding [30]. Moreover, povidone–iodine and chlorhexidine mouth rinses do not currently meet the European Standards for chemical virucidal disinfectants and antiseptics (EN 14476) since they both do not reduce the virus titer by at least four decimal logarithms (LRV ≥ 4 log10) [31]. Current COVID-19 pandemic guidelines do not recommend 1–5% povidone–iodine or 0.12–0.2% chlorhexidine in formulations for mouth rinses. Although povidone–iodine and chlorexidine are already widely used, appropriately designed in vivo studies are necessary to better assess the impact of povidone–iodine- and chlorhexidine-based preparations on oro-pharyngeal flora, tooth staining, the irritability of mucosae, and potential anosmia [17].

Furthermore, despite several antiseptics reducing SARS-CoV-2 load by 3–4 log10 in 15–30 s in vitro [17], all laboratory tests thus far have used Vero cells, which calls into question the reliability of the experiments [32]. According to the World Health organization (WHO), viral propagation in Vero cells may in fact cause genetic variants, influencing the interpretation of results from animal and clinical trials [32].

Therefore, supporting the aspecific physiological defenses of human airways against the spread of a highly mutating virus such as SARS-CoV-2 compels us to look at natural agents already part of innate defences of human airways’ mucosae.

One of these candidates proposed and tested for nasal disinfection against SARS-CoV-2 due to its intrinsic health safety is hypertonic saline solution [33]. Hypertonic saline is not directly virucidal, but NaCl seems to inactivate viral replication via depolarization of cell membrane and increased production of hypochlorous acid (HOCl) from epithelial cells of the human nasal mucosa. Hypochlorous acid, the principle ingredient of common bleach, is a disinfectant recommended by CDC, irrespective of SARS-CoV-2 variants [17]. SARS-CoV-2 replication is reportedly dose-dependently inhibited by saline solutions (0.8–1.7% NaCl) from a concentration of 0.6% NaCl, increasing up to 50% at 0.9% NaCl (isotonic saline solution) and 100% at 1.5% NaCl (mildly hypertonic saline solution) [34]. The *Edinburgh and Lothians Viral Intervention Study* (ELVIS) tested hypertonic saline nasal irrigation and gargling against other types of coronaviruses in a randomized controlled clinical trial, reporting reduction of VST by 2.6 days in patients treated with hypertonic saline lavages [35]. However, administration of nasal washings may be impractical in real life, especially to residents of a care home. Therefore, the *Regressed Nasal Infectivity and Shedding of SARS-CoV-2 by Achieving Negativization for COVID-19 Earlier* (RE.NA.I.S.S.A.N.C.E.) clinical trial recently tested in vivo the virucidal activity of an existing formulation of sweater plus additives (xylitol and panthenol and lactic acid) sprayed in the nasal cavity of patients with mild–moderate COVID-19 infected by Omicron, to reduce the respective VST. In the latter study COVID-19 patients treated with a nasal spray of seawater turned negative an average of two days earlier compared to controls if treatment was administered within the first 5 days following COVID-19 diagnosis [19].

Although saline solutions are known to be harmless, the over-production of HOCl in the nasal cavity may generate some irritation to the local epithelium in real life application. 

Another candidate considered for nasal administration against SARS-CoV-2 infection is hypothiocyanite (OSCN^−^), produced in the human airways from three components [36]:

**H_2_O_2_ + SCN^−^ → OSCN^−^ + H_2_O**
↑
**Lactoperoxidase**

- Lactoperoxidase (LPO), secreted by goblet cells and serous cells of the submucosal glands;
- Thiocyanate anion (SCN^−^), released by duct cells of submucosal gland;
- Hydrogen peroxide (H_2_O_2_), produced by epithelial cells of the airways.

A recent study tested the virucidal activity of enzyme-free OSCN^−^ against SARS-CoV-2 in vitro. In the latter experiment, enzyme-free OSCN^−^ exhibited a concentration- and time-dependent virucidal activity, slightly enhanced by the concomitant presence of lactoferrin [14]. The exact virucidal mechanism of OSCN^−^ is still unknown, but similar to high doses of ozone, the irreversible oxidative stress of lipid components of the viral envelope or nucleoproteins is likely implicated [37]. In particular, cysteine, an amino acid included in the spike protein of SARS-CoV-2, is a target for sulphydryl oxidation via OSCN^−^ [38]. At micromolar concentrations, the LPO/H_2_O_2_/OSCN^−^ system effectively proved cidal activity against a range of micro-organisms, including various bacteria (both Gram-negative as well as -positive), fungi and viruses [18,39]. Since it effectively inactivated different types of influenza virus in vitro, OSCN^−^ showed an aspecific strain-independent virucidal activity likely to be effective against any SARS-CoV-2 variants [39,40,41].

Whilst highly present in the airways epithelium, the LPO system is almost absent in the pulmonary parenchyma [42]. Aerosol administration of OSCN^−^ could eradicate an early nasal settlement of SARS-CoV-2, preventing also the downward progression of infection to the lungs [14].

However, clinical trials on humans are needed to confirm the effect of OSCN^−^ in vivo, since also the above experiment in vitro employed Vero cells [14]. A clinical trial on OSCN^−^ against SARS-CoV-2 infection should not encounter ethical issues, since the reagent is part of the physiological defences of human airways against the threat of pathogens; it already overcame a phase 1 clinical trial and did not show any cytotoxicity in vitro [14,18,38,43]. Nevertheless, in the above in vitro experiment enzyme-free OSCN^−^ was extemporaneously produced via a two-step bio-catalytic pathway, removing enzymes from the solution by ultrafiltration with a single-use dialysis micromodule. Enzyme-free OSCN^−^ is featured by a high intrinsic reactivity, thus it persists for a limited amount of time (15 minutes) in an environment, implying some limitations in real-life aerosol nasal applications [14].

N-chlorotaurine (NCT) is another natural oxidant belonging to the aspecific physiological defences of human airways, yielded from HOCl and taurine amino acid [44]:

**HOCl + Taurine → NCT + H_2_O**

Similar to OSCN^−^, NCT has a recognized broad-spectrum activity against bacteria, fungi, parasites, and viruses. The ciliary beat frequency of epithelial cells of the nasal mucosa, a very sensitive parameter for cytotoxicity, only moderately and reversibly decreased following exposure to 1% NCT, hence NCT is eligible to be applied in sensitive body districts as endogenous disinfectant [45].

Taken together, the above evidence narrows down the attention on a nasal formulation including hypertonic saline solution combined with either SCN^−^ or NCT or both, with the view of supporting the innate aspecific defences of human airways against SARS-CoV-2 and any future respiratory pathogens, responding to criteria of wide-spectrum virucidal efficacy, health safety, tolerability and cost-effectiveness.

The highly reactive HOCl, overproduced by nasal administration of hypertonic saline solution in fact oxidizes SCN^−^ into OSCN^−^ and, separately, taurine into NCT, two natural oxidants less reactive though less toxic than HOCl [14,18,46]. The nasal administration of a formulation including all three latter components could support the aspecific physiological defences of human upper airways to prevent and control the spread of any emerging SARS-CoV-2 variant in the community; however, clinical trials are needed.
